# A microcomputer-based high-speed data-acquisition system for a stopped-flow spectrophotometer: PDP-11 architecture interfaced to a commercial instrument

**DOI:** 10.1155/S1463924683000231

**Published:** 1983

**Authors:** Josephina A. Alcocer, David J. Livingston, Gerald F. Russell, Charles F. Shoemaker, W. Duane Brown

**Affiliations:** Department of Food Science & Technology University of California Davis California 95616 USA


Journal of Automatic Chemistry, Volume 5, Number 2 (April–June 1983), pages 83–88

### A microcomputer-based high-speed data-acquisition system for a stopped-flow spectrophotometer: PDP-11 architecture interfaced to a commercial instrument

**Josephina A. Alcocer, David J. Livingston, Gerald F. Russell*, Charles F. Shoemaker and W. Duane Brown**

*Department of Food Science & Technology, University of California, Davis, California 95616, USA*

### Introduction

Increasing numbers of scientists are recognizing the importance of computer control and acquisition of the large amounts of experimental data generated by modern laboratory instruments. Continued development and refinement of minicomputers and microprocessors have resulted in reductions in their cost and size, in addition to substantially higher performance. Reasonably priced computer systems mean that on-line acquisition and processing of data from different instruments is economic for many laboratories.

The technique of stopped-flow spectrophotometry has been widely used for the kinetic study of fast reactions in solution and, more recently, for the determination of chemical reactant concentrations from equilibrium methods. In these applications, data acquisition and evaluation are critical procedures. Conventionally, for reaction rate methods, data from the stopped-flow spectrophotometer are obtained as a photograph of a storage oscilloscope trace from which individual data points are extracted manually. Manual data processing is time-consuming and subject to errors from the oscilloscope photograph and the operator in transferring the data to a form suitable for calculations.

Increased use of microcomputers and microprocessors with stopped-flow systems for control, data acquisition and data processing leads to a higher level of automation with a significant increase in measurement throughput, accuracy, and precision [1]. The stopped-flow apparatus which is used routinely in many laboratories for kinetic studies is that of Gibson [2] and Gibson and Milnes [3]: this is now commercially available. One of the most widely used series of laboratory computers is the Digital Equipment Corporation (DEC) PDP-11 based machines. The recent availability of low-priced modules, such as the LSI-11 microprocessor, has made this hardware accessible to many laboratories. This paper describes an interface which can be readily used for high-speed data acquisition from the Durrum-Gibson stopped-flow spectrophotometer. This system could be readily adapted to most other stopped-flow spectrophotometers described in the literature.

### Literature

### *Reaction rates*

Reaction-rate methods of analysis may be classified into conventional and fast techniques. Conventional techniques include those where the time necessary to manually mix two reagents is usually 2 to 10 s and the mixing time must be shorter than the half-life of the reaction of interest; therefore, the reactions accessible by conventional methods are limited to first-order or pseudo-first-order rate constants of approximately 0·1 s. Fast reactions are those whose rates cannot be measured by conventional means at ordinary temperatures and concentrations [1].

The stopped-flow method uses reactants flowing in different streams through a mixing chamber and into an observation cell where a physical property of the mixed solution is measured. The use of jet mixers has reduced the mixing time to the range of 1 ms. This method has a wider reaction time range than other flow methods, with typical values of 1 to 50 ms. The lower limit is determined by the mixing efficiency—there must be sufficient time between stopping the flow and the absorbance measurement to allow the solution to become homogeneous. The upper limit depends on the stability of the detection system.

The stopped-flow technique has widespread importance in the measurement of reaction rates; it has recently been shown to be a valuable tool for analytical purposes. For moderately rapid reactions, stopped-flow mixing can provide analytical information in a very short time, often in a few seconds or less. Another reason for its acceptance in analytical chemistry is the extremely small volumes, as low as a few microlitres, required to obtain analytical information.

Examples of types of reactions which could not be studied without fast reaction techniques include [4]:

(1) Reactions of labile metal ions: ligand substitution, solvent exchange and electron transfer.
(2) Organic reactions: many proton transfer reactions, hydrogen bonding, and fast steps in polymerization reactions.
(3) Reactions of biological importance: haemoglobin with oxygen, helix-coil transitions in macromolecules, fast steps in enzyme reactions, photosynthesis, reactions in micelles and reduction of cytochromes [5].

Specific analytical applications of stopped-flow mixing include the determination of ATP, adrenaline, ascorbic acid, barium, cadmium, calcium, copper, cyanamide, cystein, epinephine, glucose, iron (III), L-dopa, heart and muscle lactate dehydrogenase (LDH), magnesium, mercury, norepinephrine, phosphate, strontium, thiolactic acid, zinc [1], and bilirubin [6].

### *Microcomputer interfaces*

Several approaches have been taken to evaluate kinetic data

**Corresponding author.*

obtained from fast reactions and avoid the slow manual procedures. One method has been to add a data-conversion unit to record the data in digital form suitable for later entry into a computer. Another method has been to obtain rate constants by an analogue curve-fitting method in which a curve, which in the simplest case is a single exponential decay, is derived from resistor and capacitor (RC) circuits. These are adjusted by superimposing the RC-derived curve on the experimental trace generated on an oscilloscope screen by the kinetic experiment. The rate constant is then determined from the resistance and capacitance values used. This method is fast and reproducible, but its accuracy depends on the accuracy with which the analogue circuit time constants are known, and it is not a permanent record of the experiment. Littler [7] used the analogue curve-fitting method calculating the exponential decay curve with a microprocessor, and displaying both the data collected through a 12-bit ADC and the simulated curves on a screen.

Automated stopped-flow apparatus which are applicable to routine work involving repetitive operation of the stopped-flow instrument have been built. In such systems the minicomputer must handle all of the instrument control functions as well as data acquisition, storage, and processing. Mieling and Pardue [8] described such a system which used a microcomputer (Intel Corporation's Model 8008) for control of reagent preparation and a minicomputer (Hewlett-Packard 2100A) for the scheduling of experiments, data aquisition, display and processing. The interface included a 12-bit analogue-to-digital converter (ADC) and a general-purpose interface with a programmable clock and buffered input/output data lines. Kouparris et al. [9] described an excellent design for an automated system built around a Rockwell Aim-65 microcomputer optimized for routine applications, especially equilibrium methods.

Bonnell and Defreeze [6] described a dual microcomputer-controlled stopped-flow spectrophotometer, with an Altair 8800B processor which handled operator interaction, data and

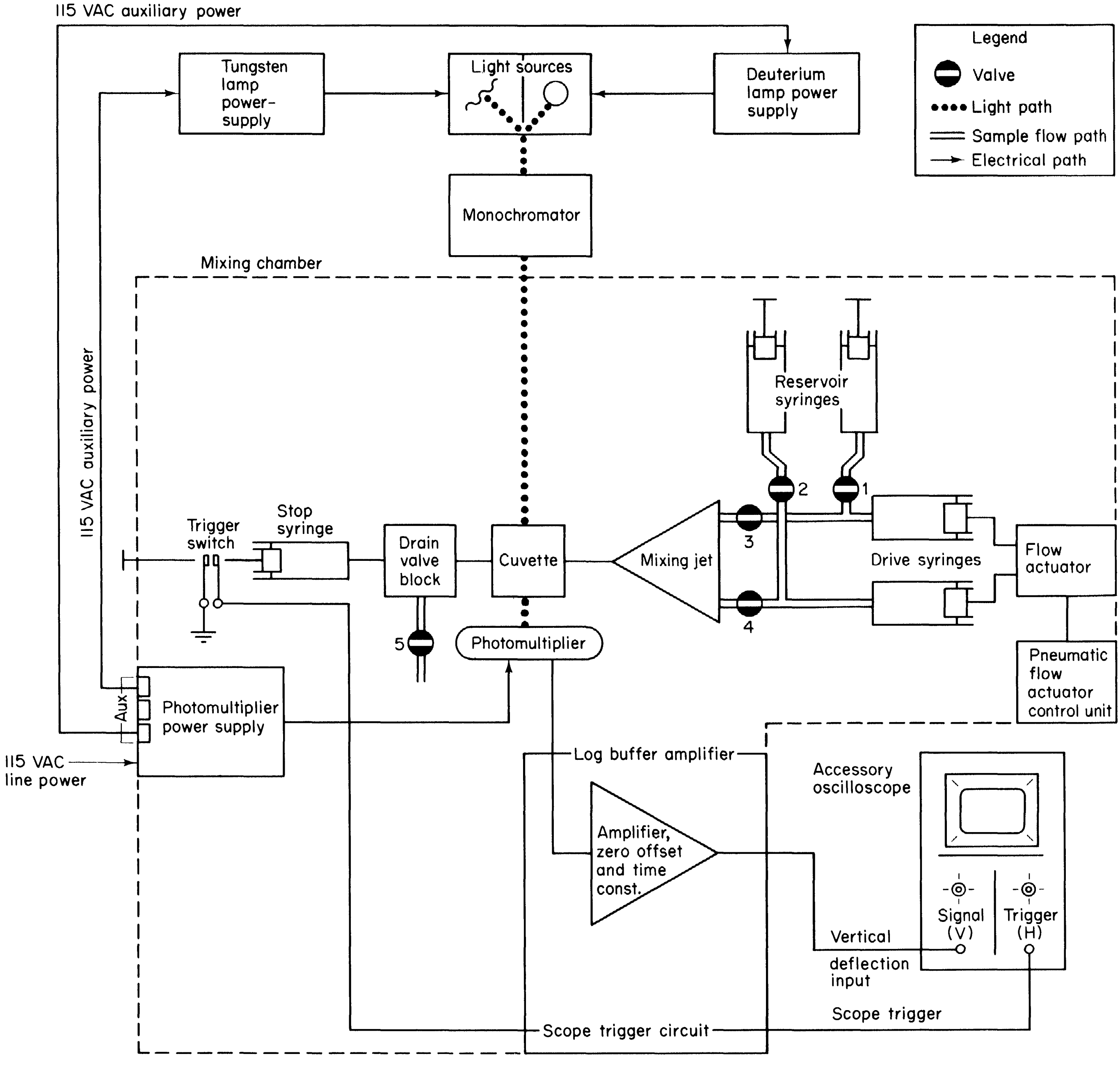

*Figure 1. Diagram of the Durrum-Gibson D-110 stopped-flow spectrophotometer (from the 'Operation and Maintenance Manual', Durrum Instrument Corporation).*

program storage and data reduction. The Altair 8800B also controlled an ADD 8080 microcomputer, which in turn controlled the operation of the stopped-flow, data acquisition, display and transmission to the Altair. Holtzman [10] calculated rates and average concentrations obtained from stopped-flow data through an Altair 8080B and a 12-bit Altair 88 ADC.

Among the researchers describing a single computer system, Zuberbuhler and Kaden [11] described an on-line data acquisition system with a Hewlett-Packard interface (HP 11203) and an HP 9820 computer. They described a non-linear least square program for calculation of rate constants. Clark and Schuster [12] interfaced a stopped-flow instrument to a Horizon II microcomputer with an 8-bit ADC. They also described a Basic program for data acquisition, storage of data files, and a plotting system for a video terminal.

The system described here combines a low-cost data acquisition module using tape storage of raw data, and compatibility with larger computers based on single-user (i.e. DEC RT-11) or time-share (i.e. DEC RSX-11M) operating systems for rapid post-run data reduction and graphics. The hardware chosen represents a good compromise between required speed, flexibility and cost, and the components are among the most common currently in use by research facilities. The software is written for use by operators with minimal training, but should allow for considerable improvement over manual methods for rapid and accurate collection and reduction of stopped-flow data.

### Instrumentation

### *Stopped-flow spectrophotometer*

A Durrum-Gibson Model D-110 stopped-flow spectrophotometer was used in this project and consisted of: (1) a mixing chamber with the sample flow system, and a pneumatic flow system which initiates the flow of reactants; (2) an optical system, which supplies monochromatic light and directs it through a sample cuvette; (3) a circulating liquid temperature-control system to maintain the flow system at a constant temperature; and (4) a storage oscilloscope, which registers and records the characteristic absorbances of the reaction. A diagram of the stopped-flow spectrophotometer is shown in figure 1.

The drive syringes are filled from the reservoir syringes, the latter having a capacity of 20 ml each. The operator actuates the system, causing equal volumes of two reactants to mix. The minimum sample size per determination is 0·15 ml of each component when using the 20 mm path-length cuvette. The solutions flow through the cuvette until the stop syringe is filled to the point where its plunger hits the mechanical stop. Flow is halted at that point so that the reaction takes place in the cuvette with a minimum of turbulence interference.

The trigger switch, actuated by the movement of the stop syringe plunger, initiates the horizontal base sweep for the oscilloscope display and also triggers the acquisition of data by the microcomputer. Light from the monochromator passes through the mixed solutions in the cuvette during the reaction and the resultant varying-intensity light is projected onto the photomultiplier tube.

The photomultiplier output, proportional to the light intensity transmitted by the reacting solution, is processed by a log buffer amplifier and drives the vertical axis of the oscilloscope. The oscilloscope display indicates transmitted light intensity versus time, starting just before the reaction begins and ending some time after the reaction has equilibrated.

### *Microcomputer and interface hardware*

The microcomputer system used for the interface was based on a 16-bit LSI-11/2 microprocessor from Digital Equipment Corporation. It was assembled on a standard DEC backplane as: (1) a Processor Module (M7270), containing the arithmetic and logic functions; (2) a Memory Module (MXV11-A2), providing 32k bytes of random access memory. This module also contains two RS-232C serial interfaces, a line-time clock, and 256 bytes of read only memory for a start-up or bootstrap program; (3) other Input Interfaces: these include an external clock and the ADC. A dual cartridge tape drive (DEC TU-58) was used as a low-cost mass-memory storage device, and provided random access to block-formatted data for data acquisition. The dual tape drive was interfaced through a serial port to the microcomputer. The data was transferred later to a disc-based system for faster and more convenient data processing.

A DEC-compatible analogue-to-digital converter (ADAC Model 1030, ADAC Corporation) was used to convert the analogue output from the stopped-flow spectrophotometer to a digital signal for the microprocessor. The ADC had a 16-channel multiplexer, a high-speed 12-bit ADC, a programmable gain amplifier, and a high-speed sample-and-hold amplifier. The external clock trigger input allowed synchronization of the ADC start pulses with an external time base. A 5 ms delay follows the trigger pulse, allowing the ADC multiplexer and amplifier to settle before conversion.

The converter uses the successive approximation technique and monolithic quad current switches to obtain stable and precise current sources that can be switched at high speeds. The reference is a zener diode with a low temperature coefficient. A high-speed sample and hold amplifier is included between the multiplexer and the ADC to reduce the aperture time during which the ADC is effectively connected to the source.

At the end of the 5 ms delay, the successive approximation conversion takes 24 ms (for the model ADAC 1030), allowing a maximum sampling rate of 35 kHz (28·5 ms per conversion) exclusive of instruction time. Faster conversion rates require a different ADC and a higher speed CPU, or direct memory access.

The programmable gain amplifier is used in applications where a dynamic range greater than 4096 (as supplied with a 12-bit converter) is needed. Four gain settings were supplied, the highest representing a ten-fold increase in sensitivity. The ADC voltage range was also board-selectable and was set $-10$ to $+10$ V for all experiments. The lowest gain was used in these experiments.

A 'Digi-Designer' (E&L Instruments) prototyping board provided a convenient apparatus to design and check temporary circuits and was used in the interface. It provides an external clock giving a TTL-compatible pulse at selectable frequencies. This clock was connected through gating circuits to the ADC external trigger to initiate the ADC conversions, each one triggered by a low-to-high pulse transition.

The Digi-Designer interface was also constructed to provide a circuit to condition the triggering signal coming from the stopped-flow spectrophotometer to the ADC trigger. The circuit, shown in figure 2, changes the stopped-flow triggering signal from approximately $-15$V to a TTL-compatible voltage acceptable to the computer. The signal from the stopped-flow trigger was connected to the clock input of a flip-flop, setting Q high, with the Q output combined through an AND gate with the Digi-Designer clock pulse, to trigger the ADC.

A Microterm 740 (ANSI standard, VT100 compatible) terminal was used as a console to communicate with the microcomputer. A Tektronix 4006-1 computer display terminal

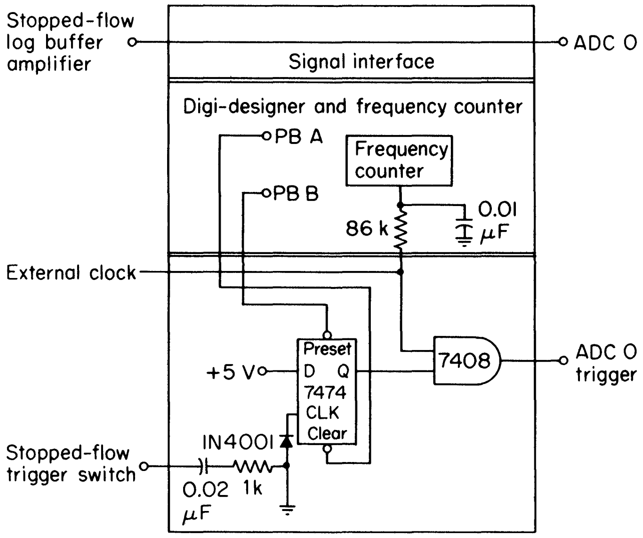

*Figure 2. Diagram of the system interface.*

(Tektronix Inc.) provided the display of the reaction curves from the processing program on the larger disc-based system after data was acquired on the cartridge tapes, and a Tektronix 4662 Interactive Digital Plotter was used as a hard-copy output device of graphic data when needed.

### Software

The software system consisted of Fortran programs, assembly language programs, and a DEC RT-11 operating system. Higher-level languages such as Fortran have slower execution speeds than programs written in assembly language. Therefore an acquisition routine (ACQ. MAC) was written in assembly language (Macro-11) for maximum execution efficiency and linked to the Fortran object module, thereby increasing the maximum rate at which data could be acquired.

### *Data-acquisition program*

The Fortran program DATA.FOR opens a file in memory storing the file name, date, time, reactants, time constant, water temperature and frequency of acquisition. It calls the macro ACQ.MAC to convert and score 1000 data points and closes the file, storing it on tape.

The macro ACQ.MAC sets the address of the peripheral device being serviced (the ADC) and initiates the service routine. The addresses for the acquired data and other necessary parameters are passed from the Fortran program to the macro through successive locations in General Purpose Register No. 5.

### *Data-processing program*

The program CONV.FOR does the kinetic calculations. It interrogates the user at the console terminal about the file name, date, gain and frequency used in the acquisition, then reads the data from the appropriate data file. The data points were originally acquired in integer form to allow the highest speed possible, but are transformed to the floating mode at this point so they can be processed and plotted.

The program plots the data on the Tektronics video screen or plotter. For this purpose, the Tektronix Plot 10 software was used (modified by R. Walraven at the University of California, Davis, USA to execute efficiently on DEC microcomputers). The absorbance data are on the $y$ axis and the point numbers (time) on the $x$ axis. An (optional) arrow points to every 20th acquisition point, to allow rapid operator interaction in selecting the first and last output points used in further calculations.

A factor, $z$, given in the ADC specifications for the voltage range used, was employed to transform the decimal value of the digitized output to voltage. With the stopped-flow calibrated for output $10\,V = 1\,OD$ (Optical Density Unit) the data in the $y$ array (in mV) are thus obtained in OD units, by the equation: $y(i) = y(i)*z/10000$. The point number on the $x$ array is transformed to a time base by dividing the points by the specific frequency used in the acquisition: $x(i) = x(i)/\mathrm{FREQ}$.

The minimum $y$ point is calculated and taken as the infinite absorbance value. The program obtains the difference between each data point and the minimum for the range chosen and takes the natural logarithm of the difference, substituting these values in the $y$ array. With these values ($y$s), and the time base ($x$s), a linear regression is made to calculate the slope of the curve of calculated first-order reaction constant, then the half-time of the reaction is obtained as $(-\ln(2)/\text{slope})$.

### Methods

### *Instrumentation*

The stopped-flow instrument was calibrated before each series of determinations was performed. A program (FLOW.FOR) to assist the user in this procedure was written to check each step in the calibration. The trigger signal from the stopped-flow instrument was connected to both the oscilloscope trigger, and the microcomputer ADC trigger. The stopped-flow log buffer output line was connected to both the vertical deflection input of the oscilloscope and the microcomputer ADC 0 input.

The program DATA.FOR was run and information on the reaction parameters entered by the user. Prior to data acquisition, PB-A of the Digi-Designer was pushed to clear the input. The stopped-flow apparatus was then actuated and the data acquired. For the examples given in the results section, Polaroid photographs of the oscilloscope display were taken and points extracted manually for comparison purposes.

### Results

### *Iron(III)-thiocyanate reaction*

To evaluate the performance of the interfaced stopped-flow spectrophotometer, one reaction monitored was the formation of the iron(III)-thiocyanate complex, which has been used as a model reaction in the stopped-flow instrument. The working solutions were $5 \times 10^{-4}$M iron(III) chloride and 0·01 M potassium thiocyanate, both diluted in 0·05 M sulphuric acid. The reaction temperature was maintained at 25°C. Willis *et al.* [13] noted that with a 0·01 M thiocyanate concentration in at least 20-fold excess over the iron (III) concentration, the reaction was first order in iron (III) concentration. The course of the reaction was monitored at 560 nm; the absorbance being proportional to the concentration of the iron(III)-thiocyanate complex.

The mechanism for the reaction is described [8] by:

$$Fe^{3+}(aq) + SCN^- \underset{k_{-1}}{\overset{k_1}{\rightleftharpoons}} FeSCN^{2+}$$

$$K_{h1} \qquad\qquad K_{h2}$$

$$Fe(OH)^{2+} + SCN^- + H^+ \underset{k_{-2}}{\overset{k_2}{\rightleftharpoons}} Fe(OH)SCN^{1+} + H^+.$$

$K_{h1}$ and $K_{h2}$ are constants for very fast hydrolysis steps. This reaction mechanism leads to a rate equation of the form [8]:

$$\frac{-d[SCN^-]}{dt} = (k_1 + k_2 k_{h1}/H^+)[Fe^{3+}][SCN^-] - (k_{-1} + k_{-2}K_{h2}/[H^+])[FeSCN^{2+}].$$

The integrated form of the rate equation is:

$$\ln\left(\frac{[FeSCN^{2+}]_\infty}{[FeSCN^{2+}]_\infty - [FeSCN^{2+}]_t}\right) = [(k_1 + k_2 K_{h1}/\ [H^+][Fe^{3+}] + (k_{-1} + k_{-2}K_{hz}/[H^+])]t$$

where $[FeSCN^{2+}]_\infty$ is the concentration of this species when the reaction reaches equilibrium. From here, we can simplify:

$$k_f = k_1 + k_2 K_{h1}/[H^+] \quad \text{and} \quad k_r = k_{-1} + k_{-2}K_{h2}/[H^+].$$

As the absorbance is proportional to the concentration of the iron(III)-thiocyanate complex at the wavelength used, the following relationship can be made:

$$\ln(A_\infty - A_t) = -(k_f[Fe^{3+}] + k_r)t + \ln \Delta A$$

where $A$ is the total change in absorbance from $t=0$ to equilibrium ($\Delta A = A_\infty - A_0$). Therefore, plots of $\ln(A_\infty - A_t)$ versus $t$ should be linear and the slope should have the form of an apparent first-order rate constant:

$$k_{obs} = k_f[Fe^{3+}] + k_r.$$

The increase in absorbance at 560 nm was recorded by the conventional oscilloscope photographic method as well as the computerized data-acquisition method. The value for the first-order rate constant was calculated as the least-square slope of the [ln($At - A$)] versus time plot. Table 1 compares the values of the calculated first-order reaction constant by the conventional method, by the microcomputer acquisition, and a reported value [13].

*Table 1. Values of calculated first-order rate constant for the iron(III)-thiocyanate complex.*

| | $k(s^{-1})$ |
|---|---|
| (*a*) Conventional method | 16·7 |
| (*b*) Microcomputer acquisition | 15·0 |
| (*c*) Reported value | 13·5 |

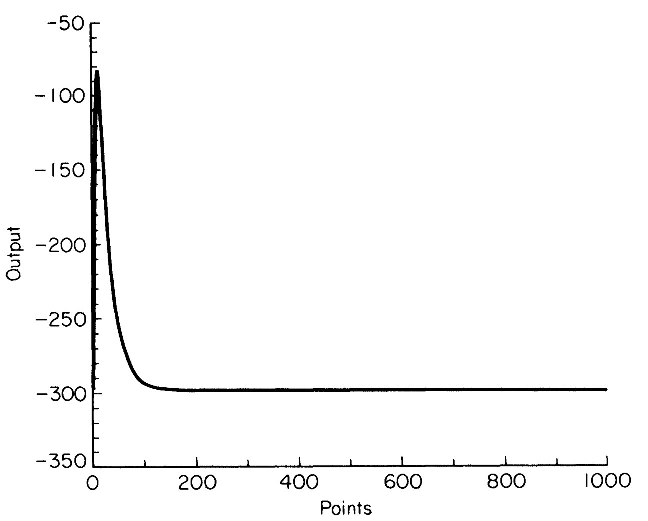

*Figure 3. Tektronix plot of digitized absorbance points versus point number (time) for the oxymyoglobin-dithionite reaction.*

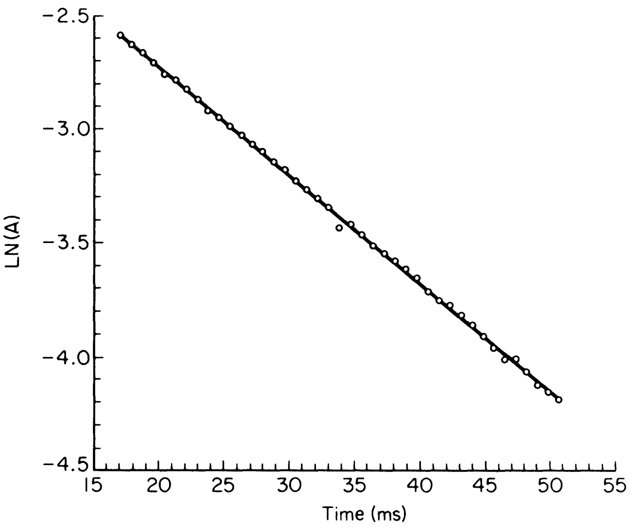

*Figure 4. Tektronix plot of ln(absorbance) versus time for the oxymyoglobin-dithionite reaction.*

### *Myoglobin reaction*

The change in absorbance of a solution of sperm whale oxymyoglobin and sodium dithionite as oxygen scavenger was monitored at 580 nm, at a reaction temperature of 25°C. The oxymyoglobin-dithionite reaction allows measurement of the velocity of dissociation of oxygen from the protein.

The results for this experiment are shown in figure 3, a plot of decimal output versus data point number, and figure 4, a semilogarithmic plot of the range of points chosen for kinetic calculations. Both figures are generated by the data reduction program on the *x-y* plotter. The calculated first-order reaction constant was 23·0 s and corresponding reaction half-time was 30·2 ms.

### Discussion

The microcomputer interface covered in this project is of the passive type, where the computer does high-speed data acquisition, but does not directly control the experiment involved. This kind of application, with the operator in charge of the calibration of the detection system, handling of reagents and start of the operation, is ideal for non-routine use of the stopped-flow instrument. Many of the biological uses for this instrument involve samples of only a few microlitres. The standard calibration procedures and determination of the correct amplification factors to bring the oscilloscope trace into range can frequently exhaust the supply of reactants. The wide dynamic range of the computerized system described here allows rapid data acquisition with assurance of valid data when only one or two experimental determinations can be done.

A minimum, low-cost system has been described. An addition that would be highly desirable in the hardware would be the use of a programmable real-time clock. At a constant acquisition rate, more data points than necessary are taken in the final part of the reaction. For the reactions monitored, the critical change in absorbance occurred in the period where the first 200 points were taken. With a programmable clock it would be possible to take points at closely spaced intervals in the region where the main change in absorbance occurs and to set larger sampling intervals at the last part—to get the maximum definition of the curve. The acquisition frequency employed was 0·5 kHz. Although the use of faster acquisition rates is possible and desirable, it would often not allow the true infinite absorbance reading (where the absorbance curve stabilizes) to be reached without dramatically increasing the size of the data buffer. This could be improved with a programmable variable rate of acquisition.

A linear regression method was used in the processing program for the calculation of kinetic data for first-order reactions. This method requires an accurate end-value or infinite absorbance reading, which in some cases is difficult to obtain. In the experimental runs recorded here, the time base for acquisition of the total number of points was compared with the time base shown in the oscilloscope display and the curve was observed to assure the accurate acquisition of an accurate end value. Otherwise, in non-routine experiments, some knowledge of the range of the reaction rate is useful.

Documented source copies of the software are available from the authors. Interested users should include a blank DEC RT-11 or RSX-11M compatible 8 in flexible disc (specify single or double density; and single or double sided), or a similarly formatted TU58 compatible Dectape-II cassette.

### References

1. Crouch, S. R., Holler, F. J., Notz, P. K. and Beckworth, P. M., *Applied Spectroscopy Reviews*, **13** (1977), 165.
2. Gibson, Q. H., *Discussions of the Faraday Society*, **17** (1954), 137.
3. Gibson, Q. H. and Milnes, L., *Biochemistry Journal*, **91** (1964), 161.
4. Caldin, E. F., *NATO Advance Study Institute Ser.*, **C50** (1979), 1.
5. Peterson, J. A. and Mock, D. M., *Analytical Biochemistry*, **68** (1975), 545.
6. Bonnell, I. R. and Defreeze, J. D., *Analytical Chemistry*, **52** (1980), 139.
7. Littler, J. S., *NATO Advanced Study Institute Ser.*, **C50** (1979), 143.
8. Mieling, G. E. and Pardue, H. L., *Analytical Chemistry*, **50** (1976), 1333.
9. Koupparis, M. A., Walczak, K. M. and Malmstadt, H. V., *Journal of Automatic Chemistry*, **2** (1980), 66.
10. Holtzman, J. L., *Analytical Chemistry*, **52** (1980), 989.
11. Zuberbuhler, A. D. and Kaden, T. A., *Chimia*, **31** (1977), 442.
12. Clark, D. D. and Schuster, S. M., *Computers and Chemistry*, **4** (1980), 51.
13. Willis, B. G., Bittikofer, J. A., Pardue, H. L. and Margerum, D. W., *Analytical Chemistry*, **42** (1970), 1340.

### NOTES FOR AUTHORS

*Journal of Automatic Chemistry* covers all aspects of automation and mechanization in analytical, clinical and industrial environments. The *Journal* publishes original research papers; short communications on innovations, techniques and instrumentation, or current research in progress; reports on recent commercial developments; and meeting reports, book reviews and information on forthcoming events. All research papers are refereed.

### Manuscripts

Two copies of articles should be submitted to the Editor. All articles should be typed in double spacing with ample margins, on one side of the paper only. The following items should be sent: (1) a title-page including a brief and informative title, avoiding the word 'new' and its synonyms; a full list of authors with their affiliations and full addresses; (2) an abstract of about 250 words—this should succinctly describe the scope of the contribution and highlight significant findings or innovations; it should be written in a style which can easily be translated into French and German; (3) the main text with sections and subsections numbered; (4) appendices (if any); (5) references; (6) tables, each table on a separate sheet and accompanied by a caption; (7) illustrations (diagrams, drawings and photographs) numbered in a single sequence from 1 upwards and with the author's name on the back of every illustration; captions to illustrations should be typed on a separate sheet.

### References

References should be indicated in the text by numbers following the author's name, i.e. Skeggs [6]. In the reference section they should be arranged thus:

to a journal

Manka, D. P., *Journal of Automatic Chemistry*, **3** (1981), 119.

to a book

Malmstadt, H. V., in *Topics in Automatic Chemistry*, Ed. Stockwell, P. B. and Foreman, J. K. (Horwood, Chichester, 1978), p. 68.

### Illustrations

Line diagrams are preferred to photographs. Original copies of diagrams and drawings should be supplied, and should be drawn to be suitable for reduction to the page or column width of the *Journal*, i.e. to 85 mm or 179 mm, with special attention to lettering size. Photographs may be sent as glossy prints or as negatives.

### Proofs and offprints

The principal or corresponding author will be sent galley proofs for checking and will receive 50 offprints free of charge. Additional offprints may be ordered on a form which accompanies the proofs.

*Manuscripts should be sent to the Editor: Dr Peter B. Stockwell, Plasma-Therm Ltd, Unit 3, 2/3 Kangley Bridge Road, Lower Sydenham, London SE26 5AR.*

